# A Hybrid Implementation-Effectiveness Study of a Community Health Worker-Delivered Intervention to Reduce Cardiovascular Disease Risk in a Rural, Underserved Non-Hispanic Black Population: The CHANGE Study

**DOI:** 10.1177/08901171221078272

**Published:** 2022-04-14

**Authors:** Carmen D. Samuel-Hodge, Ziya Gizlice, Sallie D. Allgood, Audrina J. Bunton, Amber Erskine, Jennifer Leeman, Samuel Cykert

**Affiliations:** 1 Gillings School of Global Public Health, Department of Nutrition, Center for Promotion and Disease Prevention, 144822 University of North Carolina at Chapel Hill, Chapel Hill, NC, USA; 2 Center for Health Promotion and Disease Prevention, Chapel Hill, NC, USA; 3 School of Nursing, 15776 Duke University, Durham, NC, USA; 4 Cecil G Sheps Center for Health Services Research, Chapel Hill, NC, USA; 5 School of Nursing, 144822 University of North Carolina at Chapel Hill, Chapel Hill, NC, USA; 6 School of Medicine, Division of General Medicine and Clinical Epidemiology, 144822 University of North Carolina at Chapel Hill, Chapel Hill, NC, USA

**Keywords:** lifestyle intervention, lay health advisors, rural health services, prevention and control, Non-Hispanic Black

## Abstract

**Purpose:**

To evaluate the implementation and effectiveness of the Carolina Heart Alliance Networking for Greater Equity (CHANGE) Program, an adapted evidence-based cardiovascular disease risk reduction intervention delivered by Community Health Workers (CHW) to rural adults.

**Design:**

Hybrid implementation-effectiveness study with a pre–post design.

**Setting:**

North Carolina Federally Qualified Health Center and local health department in a rural, medically underserved area.

**Sample:**

Participants (n = 255) included 87% Non-Hispanic Black with a mean age of 57 years; 84% had diagnosed hypertension, 55% had diabetes, and 65% had hypercholesterolemia.

**Intervention:**

A CHW-delivered, low-intensity, 4-month behavioral lifestyle intervention promoting a southern-style Mediterranean dietary pattern and physical activity.

**Measures:**

We measured number and representativeness of participants reached and retained, intervention delivery fidelity, weight, blood pressure, and self-reported dietary and physical activity behaviors.

**Analysis:**

Pre–post changes at 4 months were analyzed using paired t-tests.

**Results:**

Study participants completed 90% of planned intervention contacts; 87% were retained. Intervention delivery fidelity measures showed participants receiving a mean of 3.5 counseling visits, 2.7 booster calls, and on average completing 1.7 modules, setting 1.8 goals, and receiving 1.3 referrals per visit. There were significant mean reductions in systolic (−2.5 mmHg, P < .05) and diastolic blood pressure (−2.1 mmHg, P < .01); the proportion of participants with systolic blood pressure <130 increased by 7 % points (P = .05), and diastolic pressure <80 by 9 percentage points (P < .01). Dietary behaviors improved significantly with average weekly servings of nuts increased by .5 serving (P < .0001), and fruits and vegetables by .8 daily serving (P < .0001). Physical activity also increased on average by 45 min./week (P < .001). Weight did not change significantly.

**Conclusions:**

The CHANGE program showed both implementation and program effectiveness and adds to the evidence supporting CHW-delivered lifestyle interventions to reduce CVD risk among rural, Non-Hispanic Black, and medically underserved populations.

In the US cardiovascular disease (CVD) is the leading cause of death, with the greatest CVD burden concentrated in the southeastern states.^1,2^ Within this geographic region, CVD rates are highest among those living in rural communities, those with lower socioeconomic status, Non-Hispanic Blacks, and Native Americans.^3,4^ Factors contributing to CVD risk in rural communities include consuming fewer fruits and vegetables,^5,6^ engaging in less leisure-time physical activity,^7^ having higher rates of obesity, and having limited access to healthcare,^8,9^ relative to the US population as a whole. Economic burdens such as lack of insurance and higher rates of unemployment among rural residents also make it difficult to afford health care. In North Carolina (NC), approximately 4 million people, or about 40% of the population, live in one of the state’s 80 rural counties.^10^ Despite the increased risk for CVD in rural Americans, few CVD risk reduction interventions are available for rural populations.^11-13^ This represents a missed opportunity to reduce health disparities by improving modifiable lifestyle behaviors among high-risk individuals.

While there has been some CVD risk reduction intervention research in rural settings,^14-16^ evidence gaps remain for studies with Community Health Workers (CHWs) among rural Non-Hispanic Blacks. Moreover, we know little about the effectiveness of adapting and implementing CHW-delivered interventions in rural settings, especially among underrepresented populations such as Non-Hispanic Blacks. To address this gap, we developed the Carolina Heart Alliance Networking for Greater Equity (CHANGE) intervention.^17^ The CHANGE intervention adapts an evidence-based behavioral lifestyle intervention to reduce CVD risk^18^ to be delivered by Community Health Workers (CHWs), defined as frontline public health workers who are trusted members of and/or have an unusually close understanding of the community served.^19^ Although CHWs are recommended for delivery of interventions to prevent cardiovascular disease, there is insufficient evidence to guide implementation of CHW interventions in rural, medically underserved areas. We initially tested the feasibility of the CHANGE intervention in one rural, predominantly African American county.^20^ The purpose of the CHANGE feasibility study was to assess implementation and effectiveness outcomes with a goal of identifying key refinements needed for more effective implementation of the CHANGE program in a larger study sample. Here, we report on the findings from this larger study testing the CHANGE intervention’s implementation and effectiveness in a sample of high-risk adults living in a second rural, predominantly African American county with a high rate of CVD mortality.

## Methods

Design: We evaluated the implementation and effectiveness of the CHANGE program using a type 3 hybrid implementation-effectiveness design,^21^ and a single arm, pre-/post-study design. In a type 3 hybrid design, the primary research aim is to determine the impact of an implementation strategy, with a secondary aim of assessing clinical outcomes associated with implementation. Type 3 hybrid designs are used in implementation studies when there are concerns that the intervention may not be delivered with fidelity in real world settings. In CHANGE, this hybrid design fits with the focus on implementation of an evidence-based intervention adapted for CHW delivery in rural public health settings and populations.

The University’s Non-Biomedical Institutional Review Board (IRB) approved and monitored the study. This phase of the study was approved in January 2016 and direct interaction with study participants ended in August 2019. Participants were recruited from a Federally Qualified Health Center (FQHC) and Local Health Department, and all participants provided written informed consent. Participants enrolled at the FQHC also provided consent for study staff to obtain CVD-related lab values from their medical record by signing a separate Health Insurance Portability and Accountability Act (HIPAA) consent form. No medical record information was gathered for participants enrolled at the local health department.

The CHANGE Intervention: Details of the CHANGE intervention have been previously published^18^ and are briefly described here and in Table 1. Three CHWs delivered an adapted version of the evidence-based Heart-to-Health lifestyle intervention^18^ and referred participants to community and clinical resources. During the first year of study, we engaged community stakeholders in adapting the intervention to fit an underserved, rural, and predominantly African American population. As reported elsewhere,^17^ we adapted the intervention to fit delivery by CHWs who would be hired from the community, developed workflows for participant identification and referrals, and recruited local experts to co-deliver the staff training. Adaptations to the participant materials included the following: reducing and simplifying the educational text, highlighting low-cost options, adding photos of African American men, replacing some photos with local foods, and including an inventory of local resources (locations, hours, costs, and contact information).^17^

**Table 1.** CHANGE Program Content.^a^

Counseling sessions—4 Monthly Sessions (45–60 minutes duration)^b^	
Module (topic)	Topic areas
**Taking medications**	⁃What you should know ⁃Reasons for not taking medication and ways to address ⁃Local pharmacies
**Stopping smoking**	⁃What works (QuitlineNC, asking for support, medicines to help you quit)
**Healthy eating**	⁃Nuts, oils, dressings, and spreads ⁃Vegetables, fruits, beans, and whole grains ⁃Drinks, desserts, snacks, and eating out—fish, meat, dairy, and eggs
**Physical activity**	⁃Walking ⁃Keep walking and moving more ⁃Stay on track ⁃Add muscle strengthening
**Booster Calls—**3 calls after the first 3 visits (10–15 minutes duration)^b^	
**Call content**	⁃Check-in on goal progress (successes and challenges related to the topic(s) covered) ⁃Check-in on referrals (actions taken or barriers to following through) ⁃Next counseling session reminder or scheduling

^a^Four monthly counseling visits were delivered by individual in-person home visits or visits at a community location selected by the participant. A booster call was scheduled 10–14 days after each of the first 3 counseling visits.

^b^Counseling session modules are ordered by highest to lowest potential to reduce CVD risk. No more than 2 module topics (and 1 area within each topic) were covered at each counseling visit. Each session included goal setting and action-planning, with referrals to community resources, as needed.

CHANGE is a low-intensity (short duration with limited contacts) behavioral lifestyle intervention targeting CVD risk reduction through dietary and physical activity behavior changes, smoking cessation, and medication adherence. (See Table 1 for program content, planned duration of sessions/calls, and order of topics according to CVD risk reduction potential.) The dietary pattern promoted is a southern-style Mediterranean dietary pattern (Med-South) that includes affordable and familiar foods such as peanuts/peanut butter, vegetable oils, and adapted recipes for traditional southern foods such as hush puppies, collard greens, and barbeque. Med-south dietary goals include nuts and beans 3 times/week, at least 7 servings/day of fruits and vegetables, and less than 1 sugar-sweetened beverage/day. The physical activity recommendation is the same as that for US adults—at least 150 minutes/week.

The intervention involved 7 contacts: 4 monthly in-person counseling sessions delivered in participants’ homes or at local venues selected by the participant, and 3 brief booster calls between counseling visits. At the first counseling visit, participants received a manual with the full program content and a resource guide with information on local resources for healthy food, places to be physically active, wellness classes, and medication assistance. To maximize the potential benefits of lifestyle changes, sessions were introduced to participants based first on the participant’s selection of the behavior they most wanted to change, then on the potential CVD risk reduction expected (highest to lowest) by making the behavior change. In a typical session, the CHW covered up to 2 topics related to the participant’s top priority (e.g., healthy eating) followed by a session on another topic (e.g., medication adherence). Each session included goal setting, action planning, and referrals to community resources, as needed.

Site, CHW, and Participant Recruitment: Our study sites included an FQHC and a local health department in Edgecombe County, NC. Edgecombe is a rural county located in the northeastern region of NC, with a population of about 51 000, poverty rate of 21%, and about 58% of the population self-identified as Non-Hispanic Black.^22^ In 2021, Edgecombe County ranked in the least healthy quartile (0–25%) for health outcomes and health behaviors, among NC’s 100 counties.^23^ Moreover, the county’s age-adjusted stroke, heart, diabetes, and CVD death rates are all higher than the state rates.^24^

Inclusion criteria for enrollment included the following: living in or receiving medical care in the county, 18–80 years of age, and speaking English. Pregnant women were excluded or withdrawn, as pregnancy may account for observed changes in weight and blood pressure. The CHW at the health department recruited participants through community outreach, with recruitment tilted toward primary prevention of CVD. In contrast, the CHW at the FQHC recruited through the electronic health record system, with a focus on secondary prevention. Patients were targeted for participation if they were smokers or had uncontrolled diabetes (A1c greater than 8%), hypercholesterolemia (low density lipoprotein [LDL] greater than 130 mg/dL), hypertension (systolic blood pressure >140 or diastolic >90 mmHg), or a previous cardiovascular event. Patients with multiple CVD risks were prioritized.

Staff Training for Intervention Delivery: The research team conducted an intensive 4-day study training (estimated 20 hours) with the staff responsible for participant recruitment and intervention delivery (site supervisors and CHWs). Training sessions included the following content: study protocols, informed consent and participant confidentiality, participant recruitment, CHANGE intervention content, counseling principles (motivational interviewing and adult learning) and practice, community referral resources, and data collection methods.

Measures: Data collection included measures of both implementation and effectiveness outcomes; details have been published^20^ and are briefly described here and below. CHWs collected all participant data at counseling visits along with implementation process data related to participant recruitment and program delivery. To assess the utility of specific participant recruitment strategies, a single item asked participants how they heard about the program. Delivery fidelity measures included documentation of participant attendance, efforts used to contact participants missing program sessions/calls, session content and duration, goals set and progress made toward reaching goals, and referrals made with outcomes of referrals. Eligibility screening data used to rank health center patients by CVD risk factors were provided to the study staff through a data sharing agreement.

Implementation Outcomes: Data were collected to assess reach and delivery fidelity. Reach data included the number, representativeness (race/sex), and retention of participants. Fidelity data included number of visits completed, goals set, referrals made to community resources, and disposition of referrals given (action taken and/or services received).

Effectiveness Outcomes: Program effectiveness measures included blood pressure, weight, and self-reported dietary and physical activity behaviors. Outcome measures were collected at the first and last intervention visits. Blood pressure (BP) measurements were taken with an automated BP machine (Omron HEM-907XL, Omron Healthcare, Lake Forest, IL). Three BP measurements (reported as an average systolic and diastolic value) were taken at 1-minute intervals after the participant was seated for 5 minutes. Weight (average of 2 measures) was assessed in pounds to the nearest 10th, using an electronic scale (Seca 874, Seca, Hanover, MD). Self-reported dietary behaviors were measured with items from 2 validated brief food frequency surveys measuring dietary fat quality^25^ and estimated intake of fruits and vegetables.^26^ A single item adapted from the 2 items used in BRFSS^27^ was used to assess usual daily consumption of sugar-sweetened beverage consumption (“On an average day, how many 12-ounce servings of sugar-sweetened beverages do you drink with meals or in between meals? One regular can of beverage is 12 ounces.”). Self-reported data on physical activity behaviors was measured with the RESIDential Environment (RESIDE) survey^28^ which was previously modified then validated in a sample of low-income women with overweight/obesity.

Statistical Analysis: Baseline sample characteristics were summarized using descriptive statistics such as means, percentages, and standard deviations. Analyses of participant outcomes and pre–post changes at 4 months were conducted using paired t-tests. We also assessed for group differences between males and females, and age as an independent predictor of pre-/post-changes, after adjusting for baseline sex, diabetes, hypertension, and education. Since intervention effectiveness outcomes were secondary aims in this study, we did not use any imputation methods to account for missing values but provide a description of those lost to follow-up. All analyses were conducted using SAS Version 9.4 (SAS Institute, Cary, NC).

## Results

CHWs successfully enrolled 255 participants and the majority of participants were representative of the population at greatest risk for CVD in Edgecombe County where 58% of residents self-describe as Black or African American.^22^ Given that about 35% of patients treated by NC FQHCs^29^ self-describe as Non-Hispanic Blacks, the CHANGE study was quite successful in reaching this group. Participant characteristics presented in Table 2 show most participants were Non-Hispanic Black (87%) and female (72%), with a mean age of 57 years, and over half (55%) reported having a high school diploma or less in educational attainment. Risk factors for CVD included, 84% diagnosed with hypertension, 55% with diabetes, 65% had hypercholesterolemia, and about 20% were current smokers. The mean baseline blood pressure values were 127 mmHg systolic, and 75 mmHg diastolic; the mean weight was 225 lbs. Self-reported physical activity was 113 minutes per week, and dietary behaviors included 3.3 daily servings of fruits and vegetables, 1.6 servings of nuts weekly, and 1.2 (12 oz) servings daily of sugar-sweetened beverages. In characterizing the study sample by site, we observed a few significant differences. Compared to the health department sample, there was lower educational attainment, more men, higher baseline weight, and a larger proportion with diagnosed diabetes, hypertension, and hypercholesterolemia in the FQHC sample.

**Table 2.** Participant Characteristics.

Characteristic **(**N **= 255)**	N (%) or mean (SD)
Race/ethnicity, %	
Non-Hispanic Black	221 (87.4)
Non-Hispanic White	25 (9.9)
Other	7 (2.8)
Not reported	2 (.01)
Female, %	184 (72.2)
Age, y	57.5 (11.6)
Education, %	
High school diploma or less	141 (55.3)
some college	47 (18.4)
College degree (2-year or higher)	67 (26.3)
Current smoker, %	51 (20.0)
Diagnosed hypercholesterolemia, %	166 (65.1)
Diagnosed hypertension, %	215 (84.3)
Diagnosed diabetes, %	141 (55.3)
Physiologic and behavioral characteristic, mean (SD)	
Systolic blood pressure, mmHg (n = 254)	126.7 (19.7)
Diastolic blood pressure, mmHg (n = 254)	75.3 (13.2)
Weight, lb. (n=246)	225.2 (67.0)
**Behavioral**	
Physical activity, minutes/week (n = 253)	113.2 (186.3)
Fruit and vegetable servings/d	3.3 (2.0)
Healthy fats and nuts servings/wk	1.6 (.8)
Sugar-sweetened beverage servings/d	1.2 (.8)

**Figure 1.**
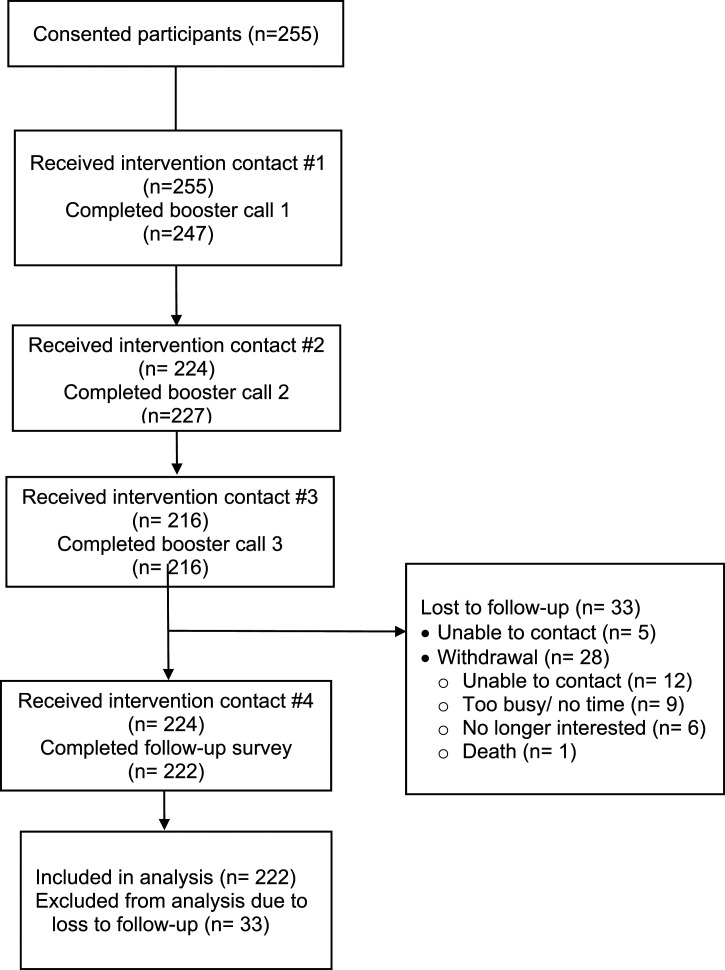
Participant flow diagram

Figure 1 shows the flow of enrolled participants through the CHANGE intervention. Among the 255 study participants enrolled, 87% (222/255) were retained, and 13% (33/255) were lost to follow-up. Those lost to follow-up included 6 participants (15%) who could not be reached, and the remaining 28 (85%) were withdrawn from the study for the following reasons: unable to contact (43%), too busy/no time (32%), no longer interested (21%), and death (4%). Participants lost to follow-up differed significantly from those included in study analysis by age and hypertension diagnosis; no race/ethnicity differences were observed. Those lost to follow-up were on average younger (52 y vs. 58 y, P < .01), and included more participants not diagnosed with hypertension (39% vs. 12%, P < .0001).

Study participants included 138 (54%) from the community health center and 117 (46%) from the local health department. The ratio of CHW to participants was 1:138 at the community health center where the CHW was full-time, and 1:58 for the two part-time CHWs at the health department.

The focus of the CHANGE study was effective *implementation* of an adapted evidence-based intervention. Figure 1, Table 2, and the text above describe our reach, representativeness, and retention. Table 3 includes other implementation variables describing recruitment strategies and intervention delivery fidelity. Nearly 60% of the 244 participants with data were recruited by a health care agency staff, site CHW, or patient lists generated from the FQHC electronic health record. Among the community-based strategies, community events (e.g., health fairs and presentations) and word-of-mouth referrals accounted for the largest proportion of enrolled participants in this category.

**Table 3.** Implementation Variables (N = 255).

Variable	Mean (SD) or N (%)^a^
Recruitment sources—How participants heard about program (n = 244)	
Site Staff	92 (37.7)
Health professional referral	4 (1.6)
Patient list (FQHC electronic health Record)	38 (15.6)
CHW	12 (4.9)
Community event	33 (13.5)
Community-based organization/church	6 (2.5)
Word-of-mouth referral	45 (18.4)
Flyer/newspaper	5 (2.1)
Other	9 (3.7)
**Program delivery**	
Counseling visits completed/participant (n = 255)	3.5 (.9)
Contact duration in minutes, mean (SD)	
Counseling visit	70.5 (91.1)
Booster call	10.2 (7.4)
Module selected as priority topic for first counseling visit, %	
Medication Adherence	15 (6)
Smoking Cessation	21 (8)
Nutrition (healthy eating)	127 (50)
Physical Activity	36 (14)
None selected	56 (22)
Mean modules completed/visit	1.7
Mean goals set/visit	1.8
Mean referrals/visit	1.3
Referral frequency by type, (%) N = 1178	
Senior or community center	68 (6)
Medication assistance	71 (6)
Smoking cessation	81 (7)
Diabetes/other support groups	61 (5)
Farmers market/food resources	236 (20)
Parks/walking or bike trail	116 (10)
Gym/walking group	94 (8)
Other community resources	451 (38)
Referrals acted on/referrals given, mean	.34
Referrals resulting in services received/referrals given, mean	.30

^a^Percentages may not add to 100 because of rounding.

Implementation of the CHANGE program, as measured by delivery fidelity, showed participants received 3.5 counseling visits on average, with average duration of 70 minutes and 2.7 booster calls on average, with average duration of 10 minutes. The proportion of planned contacts completed by each site differed slightly, with the health department participants completing 89% of all planned visits, while the health center participants completed 94%.

Participants could select the topic (module) considered their top priority and most participants (50%) chose “Healthy Eating” or did not select a priority topic (22%). Physical Activity was chosen by 14%, followed by Smoking Cessation (8%) and Medication Adherence (6%). The average counseling visit included discussion of about 2 topics and goals set, with about 1 referral made. Referrals were made to a variety of community resources, with food-related and physical activity resources accounting for the largest proportion of referrals (38% combined) among those specified. On average, participants given referrals attempted to follow-up on 34% of them and successfully accessed services for 30% of all referrals made.

Table 4 shows our program effectiveness outcomes (mean changes between pre- and post-intervention measurement) for completers. For physiological outcomes, we observed significant mean reductions in blood pressure but not weight. Moreover, there were significant increases in the proportion of participants who had a systolic pressure <130 mmHg and diastolic pressure <80 mmHg at follow-up. Self-reported dietary and physical activity behaviors also improved significantly. On average, weekly servings of nuts increased by half a serving, and fruits and vegetables by nearly one serving (.8). Participants also reported slightly lowering their daily intake of sugar-sweetened beverages. For physical activity, participants reported a mean weekly increase of 45 minutes. Analysis of differences in outcomes by sex showed no significant group differences, but some outcomes were related to age. After adjusting for baseline sex, diabetes, hypertension, and education, age was found to be a significant predictor of pre-/post-changes in systolic and diastolic blood pressure, and fruit and vegetable intake, with greater age associated with larger pre-/post-changes.

**Table 4.** Program Effectiveness Outcomes.

4-Month outcome	N^a^	Pre-Intervention Mean (SD)	Post-Intervention Mean (SD)	Mean Change (SD)	P-value
Weight, lb	215	226.6(69.2)	226.4(68.8)	−.24 (8.17)	.70
Systolic blood pressure (SBP)	222	126.9 (20.2)	124.4 (18.7)	−2.5 (17.9)	.04
Diastolic blood pressure (DBP)	222	75.3 (13.7)	73.1 (11.1)	−2.1 (11.8)	.007
Proportion at goal^a^ SBP (<130), % DBP (<80), %	222	57.7 65.8	64.4 74.8	6.7 9.0	.05 0.005
Nuts, healthy fats, servings weekly	221	1.6 (.8)	2.1 (.9)	.5 (1.0)	<.0001
Fruit and vegetable servings, daily	222	3.3 (2.0)	4.1 (1.9)	.8 (2.1)	<.0001
Sugar-sweetened beverages, servings daily	221	1.2 (.8)	1.0 (.8)	−.2 (.8)	.004
Physical activity, weekly minutes	221	114.6 (192.1)	159.9 (181.8)	45.3 (182.1)	.0003

^a^Excludes participants with missing blood pressure values at the last intervention visit.

## Discussion

Our findings demonstrate effectiveness in both implementation and effectiveness of the CHANGE intervention, and compared to our prior feasibility study,^18^ several improvements in program implementation were observed. In the paragraphs that follow, we put our results in the larger context of lifestyle interventions and CHW-delivered CVD risk reduction interventions (which are recommended by the Community Preventive Services Task Force),^13^ focus on the improvements in implementation effectiveness outcomes, and conclude with implications for future research.

CHW-delivered CVD risk reduction interventions are recommended by the Community Preventive Services Task Force,^13^ but very few studies have included rural, diverse populations. The CHANGE study helps to fill that gap. The evidence forming the basis of the Task Force recommendation came from 31 evaluations of interventions including CHWs in different roles. Most (28 studies) were conducted in the US and in urban areas (22 studies), with only 4 studies in rural settings. Thirteen studies used a pre-/post-study design without a comparison group; 23 studies included adults 18–64 years with 5 studies including older adults; and 22 studies enrolled adults from underserved groups (defined as ≥75% African American, Hispanic, or low-income). Ten studies included study populations of more than 75% female while most were evenly distributed by gender.^13^

In reviewing the studies included in the systematic review used by Community Prevention Task Force,^13^ over one-third (39%) of all studies had follow-up rates of less than 80%. Our rate of 87% points to effective training and implementation procedures. Additionally, we were able to recruit and retain a large sample of rural, Non-Hispanic Blacks, while CHWs delivered the intervention with fidelity to protocols. We also observed program effectiveness outcomes that show promise and compare favorably with similar studies. Most of the CHANGE program content focused on dietary and physical activity behavior changes. From the studies summarized in the Community Prevention Task Force report,^13^ we find that among the studies similar in design to CHANGE and delivered by CHWs, all studies reported significant improvements in nutrition and physical activity outcomes. More broadly, there is demonstrated effectiveness of lifestyle interventions among persons with CVD risk factors.^30^

A closer look at our dietary outcomes as measured by self-reported fruit and vegetable intake shows that our mean increase of .8 serving per day (an increase of 24% from baseline), compares favorably with overall outcomes of increases between .1–1.4 servings per day (or a 9.7% to 59.3% increase from baseline) reported in reviews of lifestyle interventions designed to increase adult fruit and vegetable intake.^30-33^ If maintained, our reported increase in nuts intake is similar in magnitude to that observed in the PREDIMED trial’s nut arm where increased nut intake was associated with a 28% reduction in CVD events.^34^ General improvements in healthy eating pattern by increased fruit and vegetable intake also contribute to systolic blood pressure reductions,^35^ which in turn are associated with risk reductions for major cardiovascular events and all-cause mortality.^36^ For our physical activity outcomes, comparing our mean increase of 45 minutes/week (or 40% of baseline) to other rural-based physical activity interventions,^12^ we see that among the 8 studies included in this review, only 2 reported significant differences between study arms, and relative to controls the increase in physical activity was 15%^37^ and 42%^38^ of baseline values. The 40% increase from baseline in CHANGE is an encouraging outcome. The clinical relevance of these dietary and physical activity lifestyle changes is supported by meta-analysis of data showing significant risk reduction of 18% in fatal cardiovascular events among patients engaged in multifactorial lifestyle interventions.^39^

When comparing our blood pressure outcomes to studies summarized in the Community Prevention Task Force report,^13^ we find that among studies with a non-team-based care model and similar study designs (e.g., pre-/post-test without a comparison group), our outcomes are comparable or better. Defining blood pressure goal as < 130 for systolic and <80 for diastolic pressure, we observed with a 4-month intervention, a 6.7 (systolic) and 9.0 (diastolic) percentage point increase in the proportion of participants with blood pressure at goal. Similar studies in the Community Prevention Task Force report^13^ found increases of 1.6 and 4.5 % points among studies that were 6 months or longer in duration. Our 4-month improvements in blood pressure control were much better by comparison. Changes in mean systolic and diastolic pressure in CHANGE were comparable to those reported by studies with a control or comparison group in the Community Prevention Task Force report.^13^ Our decrease of 2.5 mmHg in systolic pressure was similar to the median 2.2 mmHg decrease reported (5 studies). Two studies similar to CHANGE found an increase of 2.3 and a decrease of 3.9 mmHg. For diastolic pressure, our 2.1 mmHg decrease is better than the median decrease of 1.3 mmHg reported by 5 studies with a control or comparison group, and an increase of .5 mmHg change in 1 study of similar design to CHANGE.

With the CHANGE study primarily focused on assessing the impact of implementation strategies, after the feasibility study we targeted refinements in 3 areas for this study: pre-implementation planning for staff turnover, CHW and supervisor training improvements, and integrating CHWs into the healthcare delivery team. Our pre-implementation refinements included hiring 2 part-time CHWs (20 hours/week) for the health department site rather than 1 full-time CHW (40 hours/week), to allow for better coordination of task coverage when a CHW took leave time or turned over. We also trained one of our University study staff to deliver the CHANGE program in the event of an emergency. Improvements in staff training involved the following: (1) refining the training content and approaches, (2) adding training specifically for the CHW supervisor, and (3) providing on-going training in the form of monitoring and feedback related to implementation and program delivery. These improvements were accompanied by full retention of our CHW staff.

Other refinements included incorporating regular site meetings between the supervisor and CHW into implementation protocols and regularly monitoring the data CHWs entered into the data management system. By improving implementation planning and staff training, we anticipated improvements in both program delivery fidelity and participant retention, and both were observed. In the feasibility study, 80% of planned contacts (counseling visits and booster calls) were completed; in this phase we completed 91% of planned contacts. Our participant retention showed even greater improvements, increasing from 72% to 87% (with lost to follow-up dropping dramatically from 28% to 13%). The last area of refinement was better integration of the CHW into the health care delivery team. At the FQHC site, the full-time CHW was directly engaged with the primary health care providers and behavioral health staff. With CHW supervision by a Care Coordination supervisor, there were more opportunities for the CHW and the health care delivery team to work together in patient referrals and follow-up.

The strengths of this study are represented by these improvements in both implementation and program delivery outcomes stemming from our ability to successfully refine the strategies used in the feasibility study. This improved study helps to fill a gap in CVD-risk reduction intervention research involving CHWs among rural, Non-Hispanic Black, and medically underserved populations. Moreover, in this study with a rural sample of mostly Non-Hispanic Black adults, we had very high rates of program uptake and study participant retention. One limitation of the initial feasibility study that remains in this study is that of our study design. With a single arm, pre–post study design we are limited in attributing the observed program effectiveness to the intervention itself. As previously mentioned, our focus was on implementation because the CHANGE intervention was adapted for CHW-delivery from an evidence-based intervention. Another limitation is the short study duration relative to behavior change outcomes. Without a follow-up maintenance phase, there is no evidence of how long improvements in behaviors or blood pressure were maintained. To this point, the Task Force identified as an evidence gap, evidence from programs evaluated over periods longer than 12 months.^13^ Lastly, even though adults 18–80 years of age were eligible, we observed that those willing to volunteer were predominantly female and midlife in age. While we did not see any differences in program effectiveness by sex, the recruitment of men and younger adults to lifestyle intervention studies is area for improvement in future studies.

In summary, this study demonstrated that refinements to our initial implementation of the CHANGE program produced improvements in both implementation and program effectiveness and provide evidence supporting CHW-delivered lifestyle interventions to reduce CVD risk among rural, Non-Hispanic Black, and medically underserved adults. The potential for public health impact in CVD risk reduction and mortality is enhanced with the observed dietary changes consistent with the Southern-style Mediterranean dietary pattern promoted in the CHANGE program. Recent data from 2 large ongoing prospective cohort studies^40^ support the benefits of increasing fruit and vegetable intake on mortality risk reduction and similar health benefits are observed with intakes consistent with a Mediterranean dietary pattern.^41^ Moreover, our study findings have statewide implications for the role of CHWs and health promotion programs like CHANGE in North Carolina’s transformation of their Medicaid Program and integration of CHWs into the public health workforce while addressing social determinants of health. In July 2021, NC will begin the transformation of Medicaid from fee-for-service to managed care^42^ and with it they will include opportunities to test and evaluate the impact of addressing non-medical drivers or social determinants of health, by providing evidence-based non-medical interventions targeting housing, food, transportation, and interpersonal safety to Medicaid enrollees with high needs.^43^ A separate component of these healthy opportunities includes building infrastructure to develop and support the integration of CHWs in the healthcare workforce.^44^ Our study findings provide timely and state-specific data to inform these efforts. Beyond the NC context, there are also implications for future research targeting strategies to scale-up these low-intensity behavioral lifestyle interventions in public health agencies. Our results combining lowering of blood pressure with improved dietary habits in a hard-to-reach rural, underserved population suggest that sustaining community-clinical linkages that support chronic disease management and promote health using scalable lifestyle interventions like CHANGE could have a significant public health impact among those at highest risk.

**So What?**

**What is already known on this topic?**

Cardiovascular disease (CVD) risk reduction interventions delivered by Community Health Workers (CHWs) are recommended by the Community Preventive Services Task Force, but very few studies have included rural, racial, and ethnically diverse populations. Evidence gaps remain for implementation and effectiveness studies of CHW-delivered interventions in rural settings and among underserved Non-Hispanic Black populations.

**What does this article add?**

Using a hybrid implementation-effectiveness study design, we evaluated the implementation and effectiveness outcomes of a CHW-delivered, CVD risk reduction intervention in a sample of mostly Black adults living in a rural setting with high rates of CVD mortality.

**What are the implications for health promotion practice or research?**

Our results in a hard-to-reach rural, underserved population suggest that sustaining community-clinical linkages that support chronic disease management and promote health using low-intensity, scalable lifestyle interventions delivered by CHWs could have a significant public health impact among those at highest risk.
